# Intranasal Pharmacokinetics of Morphine ARER, a Novel Abuse-Deterrent Formulation: Results from a Randomized, Double-Blind, Four-Way Crossover Study in Nondependent, Opioid-Experienced Subjects

**DOI:** 10.1155/2018/7276021

**Published:** 2018-04-23

**Authors:** Lynn R. Webster, Carmela Pantaleon, Matthew Iverson, Michael D. Smith, Eric R. Kinzler, Stefan Aigner

**Affiliations:** ^1^PRA Health Sciences, Salt Lake City, UT, USA; ^2^Inspirion Delivery Sciences LLC, Morristown, NJ, USA

## Abstract

**Objective:**

To investigate the pharmacokinetics (PK) of Morphine ARER, an extended-release (ER), abuse-deterrent formulation of morphine sulfate after oral and intranasal administration.

**Methods:**

This randomized, double-blind, double-dummy, placebo-controlled, four-way crossover study assessed the PK of morphine and its active metabolite, M6G, from crushed intranasal Morphine ARER and intact oral Morphine ARER compared with crushed intranasal ER morphine following administration to nondependent, recreational opioid users. The correlation between morphine PK and the pharmacodynamic parameter of drug liking, a measure of abuse potential, was also evaluated.

**Results:**

Mean maximum observed plasma concentration (*C*_max_) for morphine was lower with crushed intranasal Morphine ARER (26.2 ng/mL) and intact oral Morphine ARER (18.6 ng/mL), compared with crushed intranasal ER morphine (49.5 ng/mL). The time to *C*_max_ (*T*_max_) was the same for intact oral and crushed intranasal Morphine ARER (1.6 hours) and longer for crushed intranasal morphine ER (1.1 hours). Higher mean maximum morphine *C*_max_, *T*_max_, and abuse quotient (*C*_max_/*T*_max_) were positively correlated with maximum effect for drug liking (*R*^2^ ≥ 0.9795).

**Conclusion:**

These data suggest that Morphine ARER maintains its ER profile despite physical manipulation and intranasal administration, which may be predictive of a lower intranasal abuse potential compared with ER morphine.

## 1. Introduction

Despite implementation of opioid risk management plans (including efforts to increase public awareness, guidelines for safe prescribing, clinical assessment tools, prescription drug monitoring programs, etc.), abuse of prescription opioids remains a serious public health concern. In 2016, an estimated 11.5 million individuals aged 12 years or older reported current abuse or misuse of prescription pain relievers in the United States [1]. In 2015, there were more than 15,000 deaths associated with prescription opioids (not including illicitly manufactured fentanyl or heroin) [2].

Controlled- or extended-release (ER) formulations of opioids were developed to provide patients who experience chronic pain severe enough to warrant around-the-clock opioid therapy with a consistent and sustained plasma levels of analgesic opioid. When intact tablets are taken whole, ER formulations have less appeal to abusers than their immediate-release (IR) counterparts [3]. However, because of their higher drug content per dose, ER formulations are often manipulated and administered through unintended routes. In particular, ER formulations of morphine, oxymorphone, and hydromorphone are more likely to be abused through injection because of their low oral bioavailability [4, 5]. Based on abuse rates, one study modeled the likelihood of abusing ER morphine through different routes of abuse and found that with the exception of hydromorphone, ER morphine is more likely to be abused through injection than all other opioids [4]. Manipulation of ER opioids often accelerates the release of the active opioid, essentially converting the ER opioid to an IR opioid, providing a higher blood concentration and more rapid onset of psychotropic effects compared with oral delivery as intended [6].

Abuse-deterrent formulations (ADFs) are designed to provide pain relief while deterring common methods of manipulation and reducing the potential for nonoral abuse and misuse. These formulations have properties that make nonoral abuse more difficult, less appealing, or less rewarding for one or more routes of abuse. However, these formulations are not abuse-proof, and abuse of opioid ADFs by the oral, intranasal, and intravenous routes is still possible. Clinical trials of several abuse-deterrent opioids have reported lower ratings of drug liking and positive subjective effects following manipulation compared with manipulation of nonabuse-deterrent formulations [7–9]. Furthermore, these clinical studies appear to successfully predict real-world reductions in abuse; after introduction of abuse-deterrent ER oxycodone (OxyContin, Purdue Pharma LP, Stamford, CT, USA), postmarketing data showed a decrease in rates of ER oxycodone abuse ranging from 30% to 85%, a decrease of 66% in doctor shopping for ER oxycodone, and an 85% decrease in overdose fatalities associated with ER oxycodone [10]. However, after introduction of abuse-deterrent ER oxycodone, many abusers found methods to circumvent the tamper-resistant properties or switched to other non-ADF opioids or heroin [11–14]. These data highlight the fact that opioid ADFs are one component of a comprehensive opioid risk management plan.

A novel oral ADF of ER morphine sulfate tablets (Morphine ARER, MorphaBond ER, Daiichi Sankyo, Inc., Basking Ridge, NJ, USA) resists physical and chemical manipulation, forms a viscous material if crushed and placed in liquid to prepare for injection, and retains its ER characteristics despite manipulation [15–17]. Previously published data suggest that Morphine ARER has a lower abuse potential through the intranasal route of administration when compared with ER morphine [18]. Here, we describe and compare the pharmacokinetic (PK) profile of morphine and its main metabolite morphine 6-glucuronide (M6G) for crushed intranasal Morphine ARER, intact oral Morphine ARER, and crushed intranasal ER morphine (MS Contin, Purdue Pharma, LP, Stamford, CT, USA) following administration to nondependent, recreational opioid users. Additionally, we evaluated the correlation between PK of Morphine ARER and drug liking.

## 2. Methods

### 2.1. Subjects

The selection of subjects has been detailed in a previous publication [18]. In brief, the study enrolled healthy male and female subjects aged 18 to 55 years who were nondependent, recreational users of opioids. Recreational use was defined as nonmedical use of opioids on at least 10 occasions in the past year and at least once in the preceding 12 weeks. Additionally, subjects had to have insufflated drugs at least three times in the past year. Subjects being treated for substance abuse disorder or with a history of drug or alcohol dependence were excluded.

### 2.2. Study Design and Treatment

Details of the study design have been previously reported [18]. Subjects meeting enrollment criteria first entered a qualification phase that consisted of a naloxone challenge test to exclude opioid-dependent subjects and a drug discrimination test to exclude subjects who could not distinguish the positive subjective effects of morphine from those of placebo and those who were unable to insufflate the combined volume of a 30 mg tablet of crushed morphine sulfate IR plus a crushed placebo tablet.

Qualified subjects entered a double-blind treatment period during which they were randomized to one of four treatment sequences in a four-way crossover double-dummy design, with each treatment period separated by a 7-day washout period. The following treatments were administered: crushed intranasal placebo plus oral placebo (referred to as placebo), crushed intranasal ER morphine (MS Contin) 60 mg with crushed placebo tablet added for volume plus intact oral placebo (referred to as crushed intranasal ER morphine), crushed intranasal Morphine ARER 60 mg plus oral placebo (referred to as crushed intranasal Morphine ARER), and crushed intranasal placebo plus intact oral Morphine ARER 60 mg (referred to as intact oral Morphine ARER).

The study was conducted in accord with the Good Clinical Practice Guideline (US Code of Federal Regulations, 21 CFR parts, 50, 56, and 312), the International Conference on Harmonisation (ICH), the Declaration of Helsinki, and all applicable federal and local regulation and institutional review board requirements, as appropriate. All subjects provided written informed consent to participate in the study.

### 2.3. Assessments

#### 2.3.1. Pharmacokinetics

During each treatment period, blood samples for PK assessments were obtained predose and at 0.25, 0.5, 1, 1.5, 2, 3, 4, 6, 8, 10, 12, and 24 hours postdose. A validated liquid chromatography-tandem mass spectrometry method was used to assay morphine and M6G in plasma. The calibration ranges were from 0.725 to 145 ng/mL for morphine and from 2.50 to 500 ng/mL for M6G; the lower limit of quantification for morphine was 0.725 ng/mL and for M6G was 2.50 ng/mL. Values below the lower limit of quantification were reported as 0 ng/mL.

Pharmacokinetic parameters were calculated with Phoenix WinNonlin 6.3 (Certara, Princeton, NJ, USA) using the noncompartmental model. For the PK evaluation, the following parameters were calculated using actual elapsed sampling times: maximum observed plasma concentration (*C*_max_); time associated with *C*_max_ (*T*_max_); area under the plasma concentration-time curve from 0 to 0.5, 1, 2, 8, 12, or 24 hours (AUC_0–0.5_, AUC_0–1_, AUC_0–2_, AUC_0–8_, AUC_0–12_, and AUC_0–24_); AUC from 0 to the last measurable concentration (AUC_0–*t*_); AUC from 0 extrapolated to infinity (AUC_0–∞_); elimination rate constant (*k*_e_); and apparent first-order terminal elimination half-life (*t*_1/2_). The abuse quotient (AQ; *C*_max_/*T*_max_), a PK parameter associated with drug liking and abuse potential [6, 19], was also calculated.

#### 2.3.2. Drug-Liking Bipolar Visual Analog Scale

A bipolar visual analog scale (VAS) was used to assess drug liking, the primary pharmacodynamic (PD) parameter of interest (0 = strong disliking, 50 = neither like nor dislike, 100 = strong liking). At 0.5, 1, 1.5, 2, 3, 4, 6, 8, 10, 12, and 24 hours postdose, subjects recorded their response to the question, “Do you like the drug effect you are feeling now?” by marking a single line on the VAS. The mean maximum effect (*E*_max_) and area under the drug-liking curve (AUE) for 0-1, 2, 8, 12, and 24 hours (AUE_0–1_, AUE_0–2_, AUE_0–8_, AUE_0–12_, and AUE_0–24_) were calculated [18].

### 2.4. Statistical Analysis

#### 2.4.1. Pharmacokinetic Analyses

The PK analysis population consisted of all subjects with any available *C*_max_ and AUC data. The PD population consisted of all subjects who completed all four treatment periods with at least one PD assessment in each treatment period. Sample size calculations have been described previously and were determined based on the primary PD endpoint [18].

Morphine and M6G PK parameters for each treatment were summarized for the PK population using descriptive statistics. Additionally, relative bioavailability was calculated for *C*_max_, partial AUCs (AUC_0–0.5_, AUC_0–1_, AUC_0–2_, AUC_0–8_, AUC_0–12_, and AUC_0–24_), AUC_0–∞_, and AUC_0–*t*_ using the ratio (and 90% confidence interval (CI)) of geometric means for morphine and M6G. The SAS statistical software (Version 9.2 or higher; SAS Institute, Cary, NC, USA) mixed-effect linear model procedure (PROC MIXED) was used to construct the analysis of variance models of log_e_-transformed values for each PK parameter. The model included terms for sequence, period, and treatment as fixed effects and subjects nested within sequences as a random effect. Ninety percent CIs for the difference (test minus reference) in the mean between treatments were constructed for the log_e_ scale values of each parameter. Confidence intervals were based on the least squares (LS) means estimation using the mean square error from the analysis of variance models. Least squares geometric means and 90% CIs were provided for each treatment and treatment comparison.

#### 2.4.2. Pharmacokinetic/Pharmacodynamic Analyses

Pharmacokinetic/pharmacodynamic analyses dose-response curve plots for drug liking that showed the logarithmic regression lines and coefficient of determination (*R*^2^) using logarithmic regression and the means of each parameter and treatment were created for the following: *E*_max_ versus *C*_max_ and *T*_max_ and AQ; all AUE parameters versus *C*_max_, *T*_max_, and AQ; AUE_0–1_ versus AUC_0–1_; AUE_0–2_ versus AUC_0–2_; AUE_0–8_ versus AUC_0–8_; AUE_0–12_ versus AUC_0–12_; and AUE_0–24_ versus AUC_0–24_.

## 3. Results

### 3.1. Subjects

Forty-eight subjects entered and passed the naloxone challenge; of these, 27 passed the drug discrimination test and entered the treatment phase. Twenty-five subjects completed the treatment phase [18]. PK data for intact oral Morphine ARER, crushed intranasal Morphine ARER, and crushed intranasal ER morphine, were available for 26, 26, and 27 subjects, respectively. All intranasal doses were 100% insufflated as confirmed by intranasal check.

The demographics profile of the subjects has been described previously [18]. The mean age was 25.4 years, and a majority were male (85.2%), white (96.3%), alcohol users (88.9%), and tobacco users (74.1%). All subjects had used opioids recreationally in the past 12 weeks (mean of 12.1 times for men and 9.0 times for women).

### 3.2. Intranasal Pharmacokinetics

#### 3.2.1. Morphine

**Figure 1:**
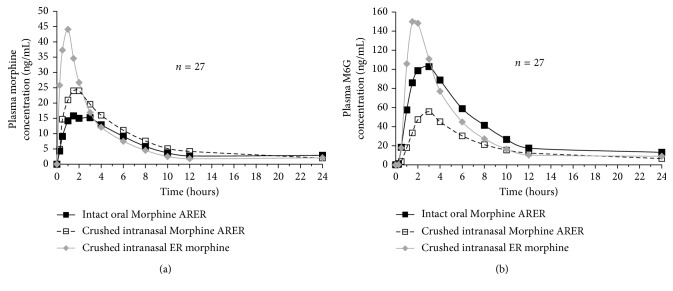
Mean plasma concentration-time profile of (a) morphine and (b) M6G by treatment (PK population, *n*=27).

The mean maximum plasma morphine concentration (*C*_max_) was lower for crushed intranasal Morphine ARER (26.2 ng/mL) and intact oral Morphine ARER (18.6 ng/mL) compared with crushed intranasal ER morphine (49.5 ng/mL) (Figure 1(a) and Table 1). Based on LS means, morphine *C*_max_ was 49% lower for crushed intranasal Morphine ARER than for crushed intranasal ER morphine (*P* value < 0.0001). Exposure to morphine at early sampling times was also lower with crushed intranasal Morphine ARER and intact oral Morphine ARER than with crushed intranasal ER morphine (Table 1). Exposure in the first 30 min (AUC_0–0.5h_) was 75% lower for crushed intranasal Morphine ARER compared with crushed intranasal ER morphine (*P* value < 0.0001). The median *T*_max_ for morphine was 46% longer (*P* value < 0.0001) for crushed intranasal Morphine ARER (1.6 hours) than for crushed intranasal ER morphine (1.1 hours).

#### 3.2.2. M6G Metabolite

As with morphine, the mean *C*_max_ for M6G was lower with crushed intranasal Morphine ARER (58.2 ng/mL) and intact oral Morphine ARER (108.2 ng/mL) compared with crushed intranasal ER morphine (169.0 ng/mL (Figure 1(b) and Table 2)). Based on LS means, M6G *C*_max_ was 68% lower (*P* value < 0.0001) for crushed intranasal Morphine ARER than for crushed intranasal ER morphine. Early exposure to M6G (AUC_0–0.5h_) was 68% lower (*P* value < 0.0001) with crushed intranasal Morphine ARER compared with crushed intranasal ER morphine. Median *T*_max_ for M6G was 94% longer for crushed intranasal Morphine ARER (3.1 hours) than for crushed intranasal ER morphine (1.6 hours).

#### 3.2.3. Morphine ARER: Intranasal versus Intact Oral Pharmacokinetics

Intranasal administration of crushed Morphine ARER resulted in slightly higher mean *C*_max_, AUC_0–5_, and AUC_0–*t*_ values for morphine compared with intact oral Morphine ARER, with similar *T*_max_ values (Table 1). However, the parameters for M6G were lower with crushed intranasal Morphine ARER, and *T*_max_ was longer compared with intact oral Morphine ARER (Table 2). Least-squares means data showed that morphine *C*_max_ was 35% higher and M6G *C*_max_ was 54% lower for crushed intranasal Morphine ARER compared with intact oral Morphine ARER. Similarly, AUC_0–0.5h_ for morphine was 43% higher and AUC_0–0.5h_ for M6G was 74% lower with crushed intranasal Morphine ARER compared with intact oral Morphine ARER. These data indicate that less morphine was metabolized to M6G within the first 30 minutes following intranasal administration of Morphine ARER. Notably, overall exposure to morphine and M6G (defined as AUC_0–*t*_ for morphine combined with AUC_0–*t*_ for M6G) was approximately 37% lower for crushed intranasal Morphine ARER compared with intact oral Morphine ARER (620.3 versus 983.8 ng · hr/mL).

### 3.3. Abuse Quotient

**Figure 2:**
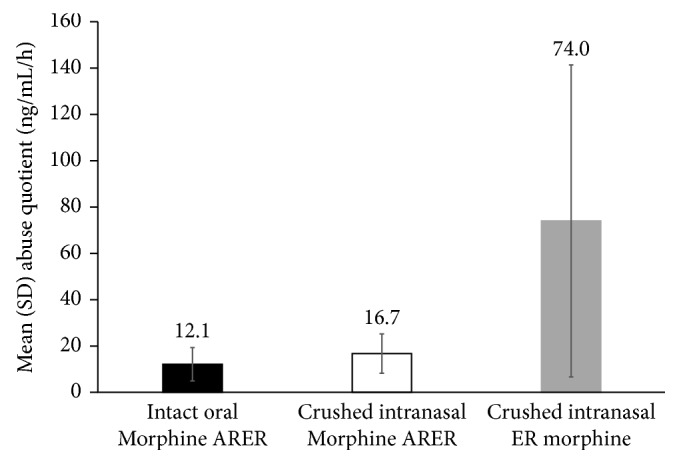
Mean (SD) abuse quotient. SD = standard deviation.

The AQ for crushed intranasal and intact oral Morphine ARER was 77% and 84% lower than that for crushed intranasal ER morphine, respectively (Figure 2). There was a large variability in the *T*_max_ for ER morphine, resulting in a wide range for AQ (15.9 to 298.7 ng/mL/hr); this variability was likely caused by the additional filler material added to blind the volumes.

### 3.4. Pharmacokinetic/Pharmacodynamic Relationships

**Figure 3:**
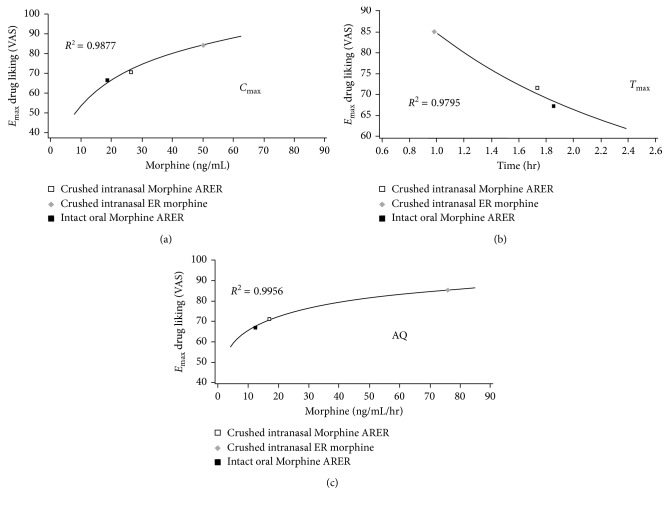
Average time-curve plots of *E*_max_ for drug liking (a) VAS versus *C*_max_, (b) *E*_max_ for drug-liking VAS versus *T*_max_, and (c) *E*_max_ for drug-liking VAS versus AQ (PD population, *N*=25). AQ = abuse quotient, *C*_max_ = maximum observed plasma concentration, *E*_max_ = mean maximum effect for drug liking, *T*_max_ = time to *C*_max_, VAS = visual analog scale.

**Figure 4:**
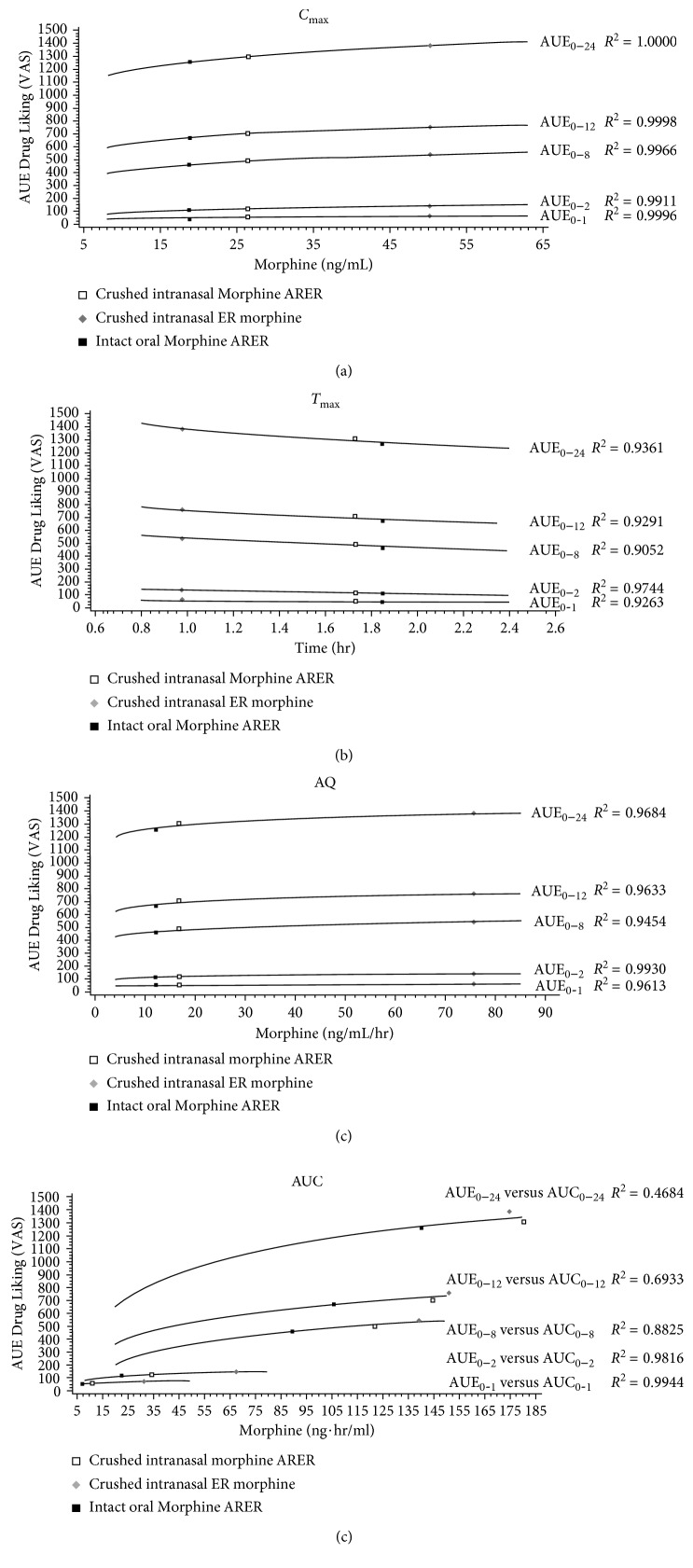
Average time-curve plot of AUE (0-1 h, 0–2 h, 0–8 h, 0–12 h, and 0–24 h) for drug-liking VAS versus (a) *C*_max_, (b) *T*_max_, (c) AQ, and (d) AUC (0-1 h, 0–2 h, 0–8 h, 0–12 h, and 0–24 h) (PD population, *N*=25). AQ = abuse quotient, AUE = area under the drug-liking curve, AUC = area under the plasma concentration-time curve, *C*_max_ = maximum observed plasma concentration, *h* = hour, *T*_max_ = time to *C*_max_, and VAS = visual analog scale.

A strong association across all active treatments was observed between mean morphine PK parameters and drug liking. *E*_max_ was well correlated with *C*_max_, *T*_max_, and AQ (Figures 3(a)–3(c)) with an *R*^2^ ≥ 0.9795 for all comparisons. A good correlation was also observed between partial AUEs and *C*_max_, *T*_max_, and AQ (*R*^2^ values ≥ 0.9911, ≥0.9052, and ≥0.9454, resp.) (Figures 4(a)–4(c)). There was strong correlation between AUC_0–1_ and AUE_0–1_ (*R*^2^ value = 0.9944) and between AUC_0–2_ and AUE_0–2_ (*R*^2^ value = 0.9816); however, the correlations became weaker over time with *R*^2^ value of 0.4684 over a 24-hour period (Figure 4(d)).

## 4. Discussion

To attain more intense euphoric effects, nonmedical users of opioids often begin abusing prescription opioids by excessive consumption of intact tablets. Abusers may progress to inhaling, injecting, or smoking the drug. Injection and insufflation are common routes of abuse for morphine [5, 20], likely because they avoid the large amount of first-pass metabolism of morphine. Data from RADARS (Researched Abuse, Diversion, and Addiction-Related Surveillance) System indicate high rates of abuse of ER morphine (both oral and nonoral) [21]. Abuse of morphine by injection and insufflation are associated with a greater risk of death or major event than ingestion by the oral route [22]. In accord with FDA guidance for evaluating abuse-deterrent opioid formulations [23], this study investigated the PK of intact oral or crushed intranasal Morphine ARER, an ER abuse-deterrent formulation of morphine, in comparison with the PK of crushed intranasal ER morphine in recreational drug abusers.

Short-term exposures to morphine and its pharmacologically active metabolite M6G were substantially lower for crushed intranasal Morphine ARER than for crushed intranasal ER morphine. Intranasal administration of crushed Morphine ARER was associated with a 49% and 68% reduction in *C*_max_ for morphine and M6G, respectively, and a 75% and 68% reduction in AUC_0–0.5_ for morphine and M6G, respectively, compared with crushed intranasal ER morphine. Crushing defeated the extended-release mechanism of ER morphine so that its PK resembled that of an IR formulation.

Crushed intranasal Morphine ARER and intact oral Morphine ARER exhibited similar PK profiles, indicating that Morphine ARER maintained its ER properties, despite physical manipulation and intranasal administration. Early exposure to morphine was slightly higher and early exposure to M6G was lower for crushed intranasal Morphine ARER than for intact oral Morphine ARER, which may be because of both the route of administration and an apparent reduction in overall bioavailability of intranasally administered Morphine ARER. At early time points, the increase in plasma morphine concentrations and the reduction in M6G concentrations compared with oral administration of intact tablets indicate that a portion of crushed intranasal Morphine ARER was directly absorbed through the nasal passage into the circulation without first-pass metabolism. Importantly, overall exposure to active morphine and M6G was reduced when Morphine ARER was crushed and insufflated compared with intact oral Morphine ARER. This reduction in overall bioavailability was likely the result of the physicochemical abuse-deterrent properties of Morphine ARER.

This study extends previous findings of reduced drug liking with Morphine ARER [18] to show strong associations between morphine PK parameters and drug liking, suggesting that the PKs of morphine from Morphine ARER are predictive of reduced drug liking and hence, of lower intranasal abuse potential. As previously reported, both crushed intranasal and intact oral Morphine ARER are associated with significantly less drug liking relative to crushed intranasal ER morphine [18]. The maximum drug-liking placebo-adjusted VAS score was 40% lower for crushed intranasal Morphine ARER compared with crushed intranasal ER morphine, and early drug-liking AUE values (over the first hour and first 2 hours) were significantly (*P* value < 0.0001) reduced for crushed intranasal Morphine ARER versus crushed intranasal ER morphine. The current PK results support these PD results, with substantially higher peak plasma concentrations in the crushed intranasal ER morphine arm, whereas crushed intranasal Morphine ARER maintained an ER PK profile that was similar to that of intact oral Morphine ARER. The lower *C*_max_ and longer *T*_max_ for morphine and M6G suggest that Morphine ARER may reduce drug liking relative to ER morphine and may be less desirable for recreational drug abusers. The AQ is a calculated parameter that captures the extent and rate of increase in plasma drug concentration as determined by *C*_max_ and *T*_max_ [6, 19, 24]. The strong correlations between AQ and drug liking support the use of AQ as a surrogate measure for drug liking. The use of AQ comparisons may be one measure to determine if a manipulated non-abuse-deterrent medication has the same abuse potential as an ADF.

Studies assessing the public health impact of an ADF of ER oxycodone (OxyContin) reported lower rates of oral and nonoral abuse for the reformulated product following its introduction [10, 12, 13, 25–27]. Across 10 studies that assessed different measures of abuse and associated consequences, the introduction of reformulated abuse-deterrent oxycodone resulted in reductions in associated oral and nonoral abuse rates, opioid use disorder, overdose, doctor shopping, and diversion [10]. However, the data also show that many users switch to other non-abuse-deterrent opioids or heroin or find methods to circumvent the tamper-resistant properties [11–14, 28]. Another ER opioid with purported abuse-deterrent properties, Opana ER, was removed from the market at the request of the FDA because of dangerous shifts in the route of abuse from intranasal to intravenous abuse [29]. This shift in route of abuse highlights the need to assess the abuse potential of opioid ADFs by all routes of abuse in both pre- and postmarketing studies. As always, it is important to remember that the currently available opioid ADFs are not abuse-proof and that abuse by the oral, intranasal, and intravenous routes is still possible.

Widespread availability and adoption of opioid ADFs by prescribers, combined with significantly reduced availability or even elimination of non-ADFs of opioids could potentially temper the rates of abusers switching to non-ADFs of opioids. Collecting real-world data on the value of ADFs has been challenging because of physicians' reluctance to prescribe opioids over concerns of possible misuse and abuse and lack of awareness of abuse patterns and impact of ADFs, underscoring the need to disseminate knowledge about prescription drug abuse [20, 30, 31].

Because of the higher cost of branded or innovator products, insurance coverage may also be limited, creating another initial barrier to uptake of abuse-deterrent opioids [20]. The results from a recent health economic analysis model indicate that converting all opioids to ADFs would reduce abuse-related costs by $266 million and improve outcomes when considering the impact on patients and rates of diversion [32].

The overall impact of ADFs on the medical system is not well understood; some studies have predicted that ADFs would increase overall health-care costs by $533 million [32], whereas others have reported potential annual medical costs savings of $429 million in the United States [33] and $4.3 billion in Canada [34]. As more opioids are developed with abuse-deterrent properties, studies assessing the impact on societal cost and outcomes are essential for determining the real-world value of opioid ADFs.

Balancing the needs of patients who have chronic pain while minimizing the diversion and abuse of prescription opioids remains a health-care challenge. Because ER opioid formulations are twice as likely to result in an overdose when compared with IR formulations [35], the reductions in drug liking seen with ADFs including Morphine ARER suggest that Morphine ARER should demonstrate a real-world benefit. Ultimately, opioid ADFs represent one component of an overall public health strategy to reduce abuse and misuse while maintaining access to pain relief [36].

## 5. Conclusions

This study demonstrates that when Morphine ARER was crushed and administered intranasally or taken orally intact, exposure to morphine and its active metabolite early after administration was significantly lower compared with crushed intranasal ER morphine. Importantly, the PK profile of Morphine ARER is similar between crushed intranasal and intact oral administration indicating that the ER characteristics of Morphine ARER were maintained after manipulation and intranasal administration. A strong and consistent association was noted between morphine PK parameters and the PD endpoint of drug liking. The results of this PK study reinforce previous findings that Morphine ARER has lower potential for intranasal abuse than non-abuse-deterrent ER morphine and support the use of AQ as a surrogate measure for drug liking.

## Figures and Tables

**Table 1:** Pharmacokinetic parameters for morphine after administration of crushed intranasal Morphine ARER, crushed intranasal ER morphine, or intact oral Morphine ARER (PK population, *n*=27).

Parameter	Mean (SD)		
	Intact oral Morphine ARER	Crushed intranasal Morphine ARER	Crushed intranasal ER morphine
*C*_max_ (ng/mL)	18.6 (5.7)	26.2 (11.2)	49.5 (17.3)
*T*_max_ (hr)	1.6 (0.5–3.1)	1.6 (1.0–3.1)	1.1 (0.2–1.6)
AUC_0–*t*_ (ng · hr/mL)	139.4 (40.1)	178.5 (77.7)	171.6 (55.2)
AUC_0–∞_ (ng · hr/mL)	158.0 (21.9)^∗^	219.8 (97.4)^†^	188.0 (51.5)^‡^
*k*_e_ (hr^−1^)	0.0688 (0.0399)^§^	0.0997 (0.0649)^‖^	0.0684 (0.0583)^∗^
*t*_1/2_ (hr)	18.4 (20.0)^§^	10.8 (8.3)^‖^	21.0 (20.9)^∗^
AUC_0–0.5h_ (ng · hr/mL)	2.1 (1.3)	2.8 (1.2)	10.9 (5.2)
AUC_0–1_ (ng · hr/mL)	7.5 (3.2)	11.2 (4.8)	30.8 (12.1)
AUC_0–2h_ (ng · hr/mL)	22.6 (7.5)	34.2 (13.7)	67.0 (22.9)
AUC_0–8h_ (ng · hr/mL)	89.3 (25.4)	120.9 (48.2)	136.5 (43.4)
AUC_0–12h_ (ng · hr/mL)	105.6 (29.5)	143.2 (57.8)	148.1 (47.4)
AUC_0–24h_ (ng · hr/mL)	139.4 (40.1)	181.1 (75.9)	171.6 (55.2)

*n*=27 for crushed intranasal ER morphine and *n*=26 for both Morphine ARER treatments, except as noted: ^∗^*n*=5, ^†^*n*=19, ^‡^*n*=4, ^§^*n*=7, ^‖^*n*=22; AUC_0–0.5, 0–1, 0–2, 0–8, 0–12, 0–24_ = area under the plasma concentration-time curve from 0 h to 0.5 h, 1 h, 2 h, 8 h, 12 h, and 24 h; AUC_0–*t*_ = area under the plasma concentration-time curve from 0 h to the last measurable concentration above the lower limit of quantification; AUC_0–∞_ = area under the plasma concentration-time curve from 0 h to infinity; *C*_max_ = maximum observed plasma concentration; ER = extended release; *h* = hour; *k*_e_ = elimination rate constant; PK = pharmacokinetic; *T*_max_ = time associated with *C*_max_; *t*_1/2_ = half-life; values for *T*_max_ are medians and ranges.

**Table 2:** Pharmacokinetic parameters for morphine 6-glucuronide (M6G) after administration of crushed intranasal Morphine ARER, crushed intranasal ER morphine, or intact oral Morphine ARER (PK population, *n*=27).

Parameter	Mean (SD)		
	Intact oral Morphine ARER	Crushed intranasal Morphine ARER	Crushed intranasal ER morphine
*C*_max_ (ng/mL)	108.2 (18.2)	58.2 (30.7)	169.0 (55.0)
*T*_max_ (hr)	2.1 (1.6–4.1)	3.1 (2.1–10.0)	1.6 (1.1–10.1)
AUC_0–*t*_ (ng · hr/mL)	844.4 (146.5)	441.8 (202.0)	777.9 (156.9)
AUC_0–∞_ (ng · hr/mL)	1054.7 (154.5)^∗^	575.1 (263.5)^†^	907.7 (158.6)^‡^
*k*_e_ (hr^−1^)	0.0720 (0.0365)^§^	0.0768 (0.0448)^‖^	0.0761 (0.0384)^‡^
*t*_1/2_ (hr)	14.0 (11.4)^§^	11.9 (6.1)^‖^	11.5 (6.2)^‡^
AUC_0–0.5h_ (ng · hr/mL)	2.2 (1.3)	0.4 (0.5)	1.9 (1.3)
AUC_0–1_ (ng · hr/mL)	18.3 (6.5)	4.8 (3.1)	26.9 (13.3)
AUC_0–2h_ (ng · hr/mL)	96.6 (20.3)	35.8 (18.9)	161.4 (68.5)
AUC_0–8h_ (ng · hr/mL)	545.2 (90.1)	266.6 (124.2)	588.3 (151.5)
AUC_0–12h_ (ng · hr/mL)	659.7 (116.5)	331.8 (149.8)	660.3 (145.7)
AUC_0–24h_ (ng · hr/mL)	844.4 (146.5)	446.8 (196.5)	777.9 (156.9)

*n*=27 for crushed intranasal ER morphine and *n*=26 for both Morphine ARER treatments, except as noted: ^∗^*n*=11, ^†^*n*=14, ^‡^*n*=6, ^§^*n*=13, ^‖^*n*=19; AUC_0–0.5, 0–1, 0–2, 0–8, 0–12, 0–24_ = area under the plasma concentration-time curve from 0 h to 0.5 h, 1 h, 2 h, 8 h, 12 h, and 24 h; AUC_0–*t*_ = area under the plasma concentration-time curve from 0 h to the last measurable concentration above the lower limit of quantification; AUC_0–∞_ = area under the plasma concentration-time curve from 0 h to infinity; *C*_max_ = maximum observed plasma concentration; ER = extended release; *h* = hour; *k*_e_ = elimination rate constant; PK = pharmacokinetic; *T*_max_ = time associated with *C*_max_; *t*_1/2_ = half-life; values for *T*_max_ are medians and ranges.
